# Pilot feasibility randomized clinical trial of negative‐pressure wound therapy versus usual care in patients with surgical wounds healing by secondary intention

**DOI:** 10.1002/bjs5.49

**Published:** 2018-04-23

**Authors:** C. Arundel, C. Fairhurst, B. Corbacho‐Martin, H. Buckley, E. Clarke, N. Cullum, S. Dixon, J. Dumville, A. Firth, E. Henderson, K. Lamb, E. McGinnis, A. Oswald, P. Saramago Goncalves, M. O. Soares, N. Stubbs, I. Chetter

**Affiliations:** ^1^ York Trials Unit, Department of Health Sciences University of York York UK; ^2^ Centre for Health Economics University of York York UK; ^3^ Academic Vascular Surgical Unit Hull and East Yorkshire Hospitals NHS Trust Hull UK; ^4^ Patient and Public Involvement Group Hull UK; ^5^ Outpatient Services Hull and East Yorkshire Hospitals NHS Trust Hull UK; ^6^ Research Office Hull York Medical School Hull UK; ^7^ Division of Nursing, Midwifery and Social Work, School of Health Sciences, Faculty of Biology, Medicine and Health University of Manchester Manchester UK; ^8^ Research and Innovation Division, Central Manchester University Hospitals NHS Foundation Trust Manchester Academic Health Science Centre Manchester UK; ^9^ Leeds Wound Research Unit Leeds Community Healthcare NHS Trust Leeds UK; ^10^ Department for Tissue Viability Leeds Teaching Hospitals NHS Trust Leeds UK

## Abstract

**Background:**

Surgical wounds healing by secondary intention (SWHSI) are increasingly being treated with negative‐pressure wound therapy (NPWT) despite a lack of high‐quality research evidence regarding its clinical and cost‐effectiveness. This pilot feasibility RCT aimed to assess the methods for and feasibility of conducting a future definitive RCT of NPWT for the treatment of SWHSI.

**Methods:**

Eligible consenting adult patients receiving care at the study sites (2 acute and 1 community) and with a SWHSI appropriate for NPWT or wound dressing treatment were randomized 1 : 1 centrally to receive NPWT or usual care (no NPWT). Participants were followed up every 1–2 weeks for 3 months. Feasibility (recruitment rate, time to intervention delivery) and clinical (time to wound healing) outcomes were assessed.

**Results:**

A total of 248 participants were screened for eligibility; 40 (16·1 per cent) were randomized, 19 to NPWT and 21 to usual care. Twenty‐four of the 40 wounds were located on the foot. Participants received NPWT for a median of 18 (range 0–72) days. Two participants in the NPWT group never received the intervention and 14 received NPWT within 48 h of randomization. Five participants in the usual care group received NPWT during the study. Ten of the 40 wounds were deemed to have healed during the study.

**Conclusion:**

A full‐scale RCT to investigate the clinical and cost‐effectiveness of NPWT for SWHSI is feasible. This study identified crucial information on recruitment rates and data collection methods to consider during the design of a definitive RCT. Registration number: ISRCTN12761776 (http://www.iscrtn.com)

## Introduction

The UK National Health Service (NHS) undertakes a substantial number of surgical operations every year, with most involving an incision^1^ that is closed with sutures or staples while healing occurs (healing by primary intention). Surgical wounds, however, may dehisce or be left open intentionally to heal ‘from the bottom up’ through formation of granulation tissue (healing by secondary intention). The management of surgical wounds healing by secondary intention (SWHSI) can be challenging as wounds may remain open for many months and/or require multiple additional treatments^2^. As a result, there may be a significant financial burden to the NHS and a substantial impact on patients' quality of life.

Existing literature focuses on guidance for the management of SWHSI; however, there is little published evidence regarding SWHSI treatment (such as type and frequency of dressings used) or wound healing rates attributable to specific treatments. Systematic reviews^3^ ^4^ have identified a limited number of trials assessing the effects of various treatments. Many of the trials were small and of poor quality, and there is little evidence to suggest that any specific treatment improves wound healing. Given the limited clinical and cost‐effectiveness evidence for existing treatments, the National Institute for Health and Care Excellence in the UK has identified RCTs of SWHSI treatments as a key research priority^5^.

In recent years advanced and costly treatments, such as negative‐pressure wound therapy (NPWT), have been used increasingly as treatments for SWHSI. It is claimed that NPWT, used as part of the treatment pathway, rather than to the point of healing, may have a positive effect on wound healing by removing exudate, reducing infections and increasing tissue perfusion^6^. There is, however, limited high‐quality research evidence to support the use of NPWT. A recent systematic review^7^ assessing NPWT as a treatment for non‐abdominal SWHSI identified only two RCTs (69 total participants), both of which reported limited outcome data. Clinical and cost‐effectiveness modelling using observational data (P. Saramago *et al*., unpublished data, 2018) showed that, even after adjustment for intergroup differences, NPWT was associated with prolonged time to healing and was highly unlikely to be cost‐effective. It is estimated (P. Saramago *et al*., unpublished data, 2018) that cessation of NPWT would save the NHS between €212 and €407 (£171 and £328) million per annum (exchange rate at 2014 prices^8^: £1 = €1·24). As these findings are based on observational data, they must be interpreted with caution.

Given that NPWT is used widely for SWHSI within the NHS, high‐level evidence investigating its clinical and cost‐effectiveness is required urgently. Recruitment to NPWT trials can be difficult^9^ ^10^, so the aim of this pilot study was to assess the methods for, and feasibility of, conducting a definitive adequately powered RCT of NPWT for SWHSI.

## Methods

Full details of the trial protocol have been published elsewhere^11^ and are given in brief below. A two‐armed, parallel‐group, pilot feasibility RCT was conducted, recruiting participants from acute and community settings in Hull and Leeds, UK.

Patients receiving care for a SWHSI from Hull and East Yorkshire Hospitals NHS Trust, Leeds Teaching Hospitals NHS Trust and Leeds Community Healthcare NHS Trust were screened against eligibility criteria by the direct care team (*Table* 1). Potentially eligible participants were provided with an information sheet to consider for at least 24 h before being approached by the study research nurse for written informed consent.

**Table 1:** Inclusion and exclusion criteria

Inclusion criteria
Aged 18 years and over and able to give full informed consent
A SWHSI that could be reasonably treated with NPWT or wound dressings
A SWHSI that was considered ready for NPWT (minimum 80% viable tissue or thin layer of slough requiring no further debridement)
Receiving adequate nutrition (as assessed by senior nurse responsible for nursing care)
Exclusion criteria
Unable to give informed consent
With limited life expectancy (for example undergoing end‐stage palliative care)
Presence of an active systemic infection
Had already received NPWT on current SWHSI
Currently receiving NPWT or received NPWT while in theatre for the operation resulting in their SWHSI
Inadequate haemostasis or at risk of bleeding
Chronic wound (such as pressure or foot ulcers) that was non‐surgical in origin but that had been surgically debrided (a distinctly different subgroup)
Unwilling to have wound photographs taken
Current or previous (in the previous 4 weeks) participant in a research study
Presence of any of the following wound characteristics
Unclear undermining in the wound cavity precluding use of NPWT
Necrotic tissue or eschar
Malignant tissue
Exposed blood vessels and/or organs, anastomotic sites and/or nerves (including cases where abdominal fascia was open)
Located where a vacuum seal could not be obtained (in the opinion of the treating clinician)

SWHSI, surgical wound healing by secondary intention; NPWT, negative‐pressure wound therapy.

Ethical approval for this study was granted by Yorkshire and Humber–Leeds East Research Ethics Committee (reference 15‐YH‐0307) and by the associated NHS trusts.

### Intervention group: negative‐pressure wound therapy

NPWT devices that were being employed in the acute and community services in Hull and Leeds were selected for use in this study. Eligible NPWT devices were V.A.C.® (KCI, San Antonio, Texas, USA), Renasys® (Smith & Nephew, Hull, UK) and PICO® (Smith & Nephew). The choice of device and duration of the therapy were decided by the clinical care team in conjunction with the participant. Aspects of the NPWT treatment regimen, such as pressure cycles and dressing change frequency, were according to the standard practice at the time. The only stipulation made for NPWT use was that it must be clinically appropriate. When not being treated with NPWT, participants' wounds received usual care. The type of device, dressings and pressure cycles were recorded, and any changes reported at subsequent follow‐up visits.

### Comparator group: usual care

Control group participants received usual care (wound dressings, no NPWT). The type of dressing used was not stipulated for the trial as there is no evidence to suggest that any one dressing is more clinically or cost‐effective than another^3^. The most appropriate dressings and frequency of dressing change were selected by the clinical care team and recorded throughout the trial.

### Baseline data and follow‐up visits

Participant demographics, wound and surgery characteristics, and participant self‐reported questionnaires (assessing quality of life and pain scores) were collected at the baseline visit. Where participants had more than one SWHSI, the largest wound was deemed the ‘reference’ wound, and data were collected for this wound during the study.

After randomization, participants were followed up for a maximum of 3 months unless they withdrew consent, died or were lost to follow‐up. Clinical data were collected by research nurses on a weekly to fortnightly basis at follow‐up assessment visits. The timing of clinical follow‐up was not standardized (beyond weekly to fortnightly) to allow the feasibility of visit frequency to be assessed. Visits continued, up to a maximum of three visits, following an assessment that deemed the wound to have healed.

### Feasibility

Feasibility outcomes included: eligibility rates (calculated by dividing the number of people who met eligibility criteria by the number screened for eligibility) and reasons for ineligibility; participation rates (calculated by dividing the number of participants randomized by the number of people eligible for the study) and reasons for non‐participation; withdrawal rates (calculated by dividing the number of participants who withdrew from the study by the number recruited) and the reasons for withdrawal; ability to deliver the intervention, including time between randomization and treatment; reasons for delays in start of treatment; duration of NPWT use; reasons for NPWT cessation; and treatment crossover. The need for training to deliver NPWT was assessed via a questionnaire completed by research nurses at the end of the study.

The trial protocol advised that a photograph of the wound be taken at each assessment visit. Research staff were provided with brief guidance on taking these photographs, which stipulated that the entire wound should be in view and the participant identification be included in the image. The feasibility of blinded outcome assessment of healing and treatment allocation was assessed by providing blinded assessors with copies of the wound photographs and asking them to confirm whether: they deemed the wound to be healed (full wound epithelialization); and they believed they knew the participant's treatment allocation. However, in practice it was not feasible for the blinded reviewers to assess all participant wound photographs. It was therefore decided that the last photograph taken after randomization for each participant would be used in the feasibility of blinded assessment of healing. Blinding to treatment allocation was assessed using photographs taken at week 1, as the final photograph may not reflect the originally allocated treatment given that NPWT is not necessarily used to the point of wound healing.

Finally, the feasibility of collecting wound pain data scored on a scale from 0 (no pain) to 10 (worst pain imaginable) using weekly text messages was assessed.

### Clinical outcomes

#### *Healing*

The primary clinical outcome in a definitive trial would be time in days from randomization to wound healing. Healing was assessed at each follow‐up visit by the research nurse and, when observed, the date of healing was recorded.

#### *Wound size*

The wound area at baseline and at each follow‐up visit was calculated using ruler measurements: without adjustment (length × width); and with adjustment, by multiplying the area by π/4 (length × width × 0·785)^12^. In addition, where possible, a wound tracing was taken at the same time and the area measured using Mouseyes software (R. Taylor, Manchester, UK)^13^.

#### *Health‐related quality of life and pain*

Participants self‐completed questionnaires at baseline, 2 weeks, and 1 and 3 months after randomization. Follow‐up questionnaires were posted with a stamped addressed envelope for return to the clinical trials unit. Questionnaires included two generic health‐related quality‐of‐life (HRQoL) instruments, Short Form 12 (SF‐12®; Quality Metric, Lincoln, Rhode Island, USA) and EQ‐5D‐3L™ (EuroQol Group, Rotterdam, The Netherlands), and three pain assessment instruments: a visual analogue scale (VAS) for wound pain, and a VAS for pain experienced when wound dressings were changed, both scored from 0 (no pain) to 10 (worst pain imaginable), and the nine‐item short form of the Brief Pain Inventory (BPI)^14^. The BPI comprises two subscales: average pain severity, scored from 0 (no pain) to 10 (pain as bad as you can imagine); and level of interference with mood and daily activities caused by pain, scored from 0 (does not interfere) to 10 (interferes completely).

#### *Clinical and adverse events*

Data were collected on clinical events (such as duration of hospital stay and wound infection) and adverse events (both serious and non‐serious) throughout the trial.

#### *Cost‐effectiveness*

This was explored using total mean resource use cost and mean quality‐adjusted life‐year (QALY) per trial arm.

### Sample size

As this was a pilot trial, and the primary objective was to determine measures of feasibility and acceptability rather than to detect a treatment effect, a formal power calculation was not conducted. The study aimed to randomize approximately 50 patients in order to assess eligibility, participation and withdrawal of participants.

### Randomization and blinding

Consenting participants were assigned 1 : 1 randomly to receive either the intervention (NPWT) or usual care (no NPWT). The randomization sequence was prepared by the trial statistician (who was not involved in study recruitment) using random permuted blocks of size 4 and 6 with stratification by wound area (less than 28 cm^2^ or 28 cm^2^ or more). Wound area was found to be associated with wound healing in an earlier cohort study conducted by the trial team (I. Chetter *et al*., unpublished data, 2018), in which the median wound size among patients who received NPWT was 28 cm^2^. The use of mixed block sizes, without stratification by site, reduced predictability of allocation.

Allocations were concealed through a central randomization service implemented by an independent member of staff at the York Trials Unit, University of York. Once a patient had been consented, researchers telephoned the York Trials Unit to obtain the treatment allocation. Owing to the nature of the intervention, it was not possible to blind participants or healthcare professionals to trial treatment.

### Statistical analysis

Data analysis was conducted in Stata® version 13 (StataCorp, College Station, Texas, USA) using the principle of modified intention‐to‐treat on an available case basis. As this was a pilot trial, analyses are mainly descriptive in nature, with summary statistics presented overall and by trial arm according to participants' original treatment allocation, where data are available.

Time to healing was assessed using Kaplan–Meier curves and the log rank test, recognizing that the study was not powered to detect a treatment effect, and that this was not a trial objective. Cox proportional hazards regression was used to investigate the impact of co‐variables shown to be important in an earlier SWHSI cohort, including contamination level of surgery, duration of wound in days (time from surgery that resulted in SWHSI to randomization), and presence of infection at baseline (I. Chetter *et al*., unpublished data, 2018). The stratification factor (baseline wound size), in its continuous form as opposed to being dichotomized, was also used as a co‐variable in the Cox model. The effects of SWHSI history, location of SWHSI on the body, and infection at any time during follow‐up (as a time‐dependent co‐variable) were also explored by including these, in turn, in the adjusted Cox model.

Bland–Altman plots^15^ were used to assess agreement of the measurement methods for wound size (ruler *versus* tracings) used in the study.

## Results

Recruitment commenced at Hull and East Yorkshire Hospitals NHS Trust on 20 November 2015, at Leeds Teaching Hospitals NHS Trust on 9 February 2016, and at Leeds Community Healthcare NHS Trust on 3 March 2016, and ran for a total of 7 months, closing at all sites on 30 June 2016. Follow‐up continued for a further 3 months, until 30 September 2016.

**Figure 1:**
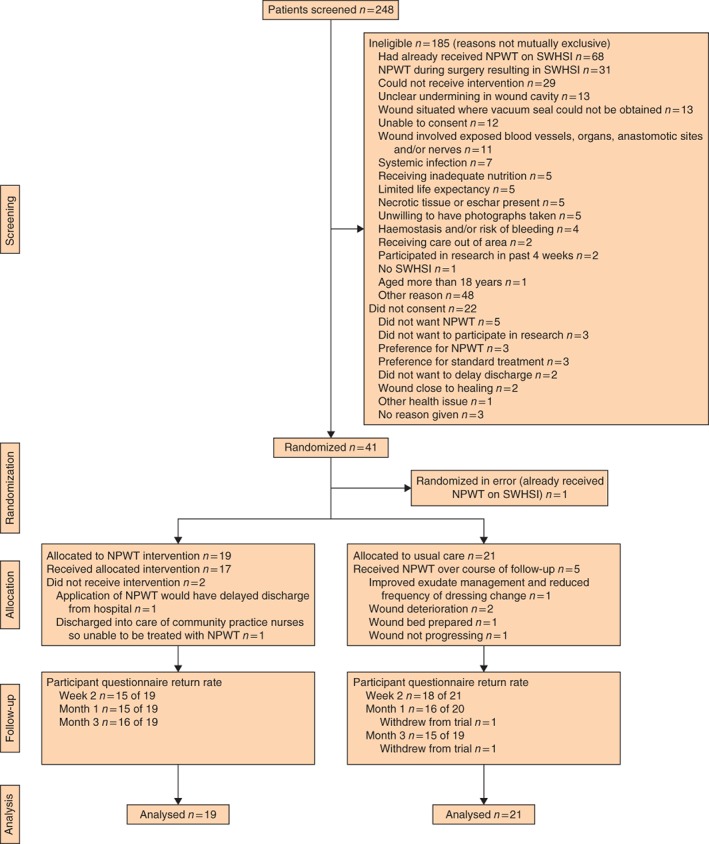
CONSORT diagram depicting the flow of participants through the trial. NPWT, negative‐pressure wound therapy; SWHSI, surgical wound healing by secondary intention

Delays in receipt of local research and development approvals were observed for two sites, owing to circumstances within the local trusts beyond the control of the research team. This affected the recruitment rate in the first 3 months of the trial; however, after the opening of all three recruitment sites, the study achieved the recruitment target (7 participants per month) in the remaining 4 months of recruitment. In total, 41 participants were randomized into the trial. One participant was subsequently found to be ineligible, as they had previously received NPWT on their SWHSI. Data for this participant were excluded from all data summaries and analysis as they were randomized in error. Thus, 40 eligible participants were randomized, 19 to NPWT and 21 to usual care. The flow of participants through the trial is shown in *Fig*. 1.

### Baseline characteristics

Baseline characteristics were similar in the two groups (Table 2). The mean age of participants was 57·8 (range 33–88) years, and 22 were men. Wounds were commonly located on the foot (24 patients); the initial indication had been partial foot or toe amputation for 20 patients, debridement of a non‐chronic wound on the foot for two, and abscess drainage for two. Thirteen wounds were reported as being infected at baseline. Twenty‐four participants had diabetes. Participants allocated to usual care tended to have had their wounds for longer than those allocated to NPWT (median 12 versus 7 days respectively), and had a higher incidence of wound infection (8 of 21 (38 per cent) versus 5 of 19 (26 per cent)), as assessed by clinicians against a predefined features list (Appendix S1, supporting information).

**Table 2:** Baseline characteristics of randomized participants by treatment group

	NPWT (n = 19)	Usual care (n = 21)	Total (n = 40)
Age (years)*	58·8(15·1)	56·9(13·9)	57·8(14·4)
Sex ratio (M : F)	11 : 8	11 : 10	22 : 18
BMI (kg/m^2^)*	29·2(5·6)	29·0(6·0)	29·1(5·7)
Diabetes	12	12	24
Peripheral arterial disease	7	7	14
Tobacco use			
None	13	10	23
Ex‐smoker	1	6	7
Current (≤ 1 pack/day)	2	4	6
Current (> 1 pack/day)	3	1	4
Previous history of SWHSI			
Yes	5	5	10
No	11	15	26
Unknown	3	1	4
Wound area (cm^2^)†	18·2 (0·2–122·5)	20·4 (2·3–93·2)	19·3 (0·2–122·5)
< 28	10	11	21
≥ 28	9	10	19
Wound duration (days)†	7 (0–27)	12 (1–142)	7 (0–142)
Wound infected			
Yes	5	8	13
No	14	13	27
Wound location			
Foot	11	13	24
Abdomen	3	3	6
Leg	3	1	4
Breast	1	1	2
Groin	0	2	2
Buttocks	0	1	1
Perianal area	1	0	1
Type of surgery‡			
Elective	12	18	30
Emergency	6	3	9
Contamination level of surgery			
Clean	6	2	8
Clean‐contaminated	9	12	21
Contaminated	4	7	11

Values are

* mean(s.d.) and

† median (range).

‡ Type of surgery not known for one patient in the negative‐pressure wound therapy (NPWT) group.

### Feasibility outcomes

#### *Eligibility rate*

Of the 248 patients screened, 63 (25·4 per cent) were found to be eligible. Common reasons for ineligibility included having already or previously received NPWT on the SWHSI (99 of 185 ineligible patients, 53·5 per cent), which was often applied in theatre at the time of surgery (31 patients), and the wound not being suitable for NPWT (29, 15·7 per cent).

#### *Participation rate*

Of the 63 eligible patients, 41 (65 per cent) consented to participate in the trial. A variety of reasons for non‐participation were observed, the most common (5 of 22 patients, 23 per cent) being that the patient did not wish to receive NPWT.

#### *Withdrawal rates and retention of participants*

Of the 40 eligible randomized participants, two were fully withdrawn from the trial due to amputation of the reference wound (at 24 and 60 days after randomization), equating to a withdrawal rate of 5 per cent (2 of 40). Both participants were allocated to usual care. The study protocol was subsequently revised to enable continued data collection from participants after amputation, provided they continued to give consent. No further participants, however, underwent amputation.

Participants received between one and 12 assessment visits after randomization (median 8 in NPWT and 7 in usual care group), with the first postrandomization assessment occurring between 4 and 33 days (median 7·5 days in NPWT and 9 days in usual care group). Overall, participant self‐completed questionnaire response rates were at least 78 per cent (31 of 40) at all time points.

#### *Ability to deliver intervention*

Of the 19 participants allocated to receive NPWT, two never received the intervention. In one patient this was because it would have delayed hospital discharge, and in the other because a NPWT device was not available in the community at the time. Ten participants received NPWT within 24 h of randomization and 14 within 48 h. The primary reason for delay (receiving NPWT 24 h or more after randomization) was the machine being unavailable (6 of the 7 remaining patients).

A Renasys® machine was applied in 12 of the 17 participants who received NPWT, PICO® in four and V.A.C.® in one patient. The pressure used ranged between 60 and 125 (median 80) mmHg, and was applied continuously in all but one patient (PICO®; 80 mmHg pressure applied intermittently). In addition, two participants treated with PICO® NPWT were reported to have had their wounds packed with Aquacel® (Convatec, Reading, UK) (hydrocolloid) and dressed with an Opsite® (Smith & Nephew) (vapour permeable) dressing.

Participants in the intervention group received NPWT for a median of 18 (range 0–72) days. Two of the 19 participants ceased NPWT therapy on the day of randomized treatment receipt due to a failure to maintain the seal. Other reasons for discontinuation of NPWT included: wound improving (8 patients) and wound healed, wound deteriorated, wound bed prepared, wound too dry, wound too small, wound edges bleeding, and blood in tubing of canister (1 patient each).

Among the 21 participants receiving usual care, hydrocolloid dressings were the most common initial treatment (11 patients). Other initial dressings included silver‐containing dressings (3 patients), iodine‐containing dressings (3), basic dressings (3) and a soft polymer dressing (1). Five participants in this group subsequently crossed over from their initial dressing to NPWT, a median of 4 (range 0–17) days after randomization. Reasons for crossing over included: improved exudate management and reduced frequency of dressing change (1 patient; NPWT commenced on day 0 after randomization), wound deterioration (2 patients; commenced NPWT on day 4 and 6 after randomization), that the wound bed was prepared for further treatments, for example skin grafting (1 patient; day 3), and that wound healing was not progressing (1 patient; day 17). These five participants remained on NPWT for a median of 31 (range 19–36) days. No other participants in the usual care group were reported to have had NPWT applied during the course of the trial.

Of the eight research nurses involved in the study, six indicated that, as part of their role, they would apply NPWT for study participants. Not all the nurses had the opportunity to do this during the trial, but of those who indicated they would have to apply NPWT to study participants, four felt they had received sufficient training, through their institutions, to apply this treatment confidently. All eight research nurses completing the questionnaire indicated that not all of their colleagues had the required knowledge and skills to use NPWT, and that specific standardized training in NPWT application would be required as part of a definitive RCT.

#### *Blinded outcome assessment of healing*

Agreement on the assessment of wound healing status (according to the final photograph taken of the wound) by two initial reviewers was achieved for 29 (73 per cent) of the 40 participants (15 in the NPWT and 14 in the usual care group). After assessment of the remaining 11 participants' final photographs by a third reviewer, agreement between two reviewers was reached for 38 participants (95 per cent), but for two (both in the NPWT group) each reviewer recorded a different judgement (healed, not healed, and unsure). Where the reviewer was unsure and a reason for this was provided (8 photographs), it was primarily due to poor photographic quality (4). Overall, eight of the 40 wounds (20 per cent) were judged by the assessors to have healed (3 in the NPWT and 5 in the usual care group); seven of these were also recorded as healed by the investigators at the weekly to fortnightly visits. For three additional wounds judged as healed by the investigators the blinded assessors were either unsure of healing status or there was discordance between all reviewers.

Assessors correctly identified treatment allocation using the week 1 photograph for 15 of the 40 participants (9 in the NPWT and 6 in the usual care group). Allocation to NPWT was identified incorrectly in six cases.

#### *Collection of wound pain data using weekly text messages*

Twenty‐six participants (65 per cent, 13 in each group) consented to be sent a weekly text message asking about their pain, and 20 provided at least one response (10 in each group). The majority of participants who responded provided at least four responses (19 of the 20 responders), and eight responded to all text messages sent. The overall mean pain score reduced between week 1 (mean(s.d.) 4·0(3·0)) and month 3 (mean(s.d.) 1·6(2·0)), although fluctuations in scores were observed during this time interval (Table S1, supporting information).

### Clinical outcomes

#### *Healing*

**Figure 2:**
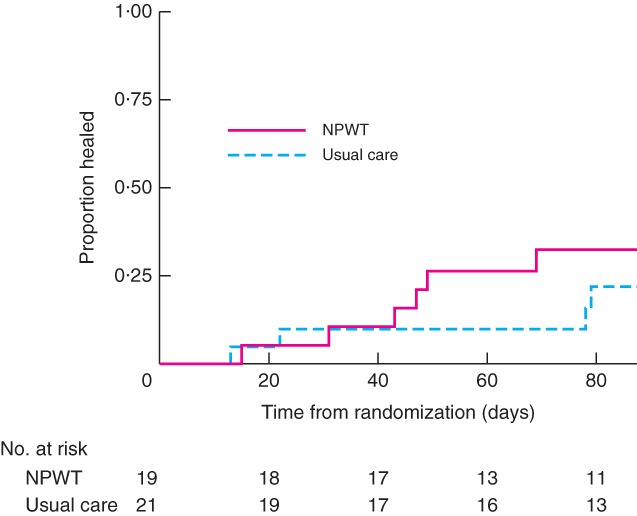
Kaplan–Meier curve of time from randomization to healing in patients undergoing surgical wound healing by secondary intention treated with negative‐pressure wound therapy (NPWT) or usual care. P = 0·418 (log rank test)

Over the course of the trial, the wounds of ten (25 per cent) of the 40 participants were deemed to have healed based on the non‐blinded research nurse assessments at the weekly to fortnightly visits (6 in the NPWT and 4 in the usual care group) (Fig. 2). Evidence from the adjusted Cox proportional hazards models suggested that wound size and duration were likely to be associated with time to healing (Table S2, supporting information).

#### *Wound size*

**Figure 3:**
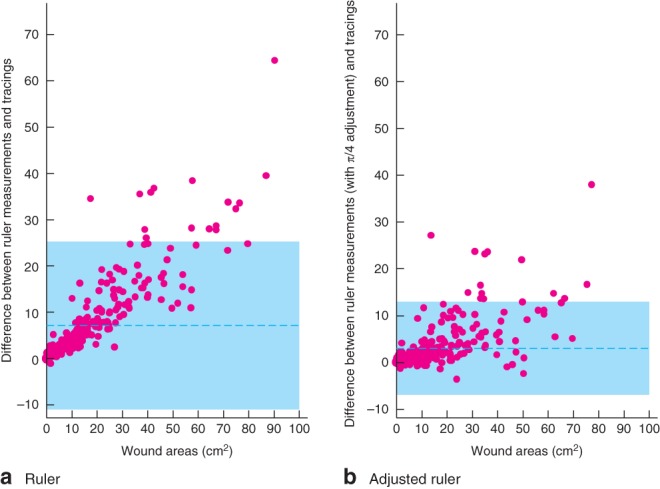
Bland–Altman plots demonstrating the agreement between measurements from wound tracings and measurements of wound area made using **a** a ruler and **b** an adjusted ruler. The difference between the two measurements made on the same observation (y‐axis) is plotted against the mean of the measurements (x‐axis), and each is represented with a dot. The shaded area depicts the 95 per cent agreement interval, within which 95 per cent of the differences fall. For example, the 95 per cent limits of agreement for plot **a** are −11·3 to 25·6, indicating that for 95 per cent of observations a ruler measurement of wound area is between −11·3 and 25·6 cm^2^ larger than a tracing measurement. This plot was produced using the batplot command in Stata®, with the notrend option

In general, wounds appeared to decrease in size in both treatment groups at a similar rate over time (*Table S3*, supporting information). The Bland–Altman plots indicate that measurement of the wound using a ruler without adjustment (length × width) overestimates wound area compared with using the tracings (*Fig*. 3 *a*). Adjusted wound area measurement (length × width × 0·785) showed better agreement with the tracings, but still tended to overestimate the area (*Fig*. 3 *b*). In both cases, overestimation tended to increase with wound size.

#### *Quality of life and pain*

Improvement in the mean SF‐12® Physical Composite Scale (PCS) score was observed over time in both groups. The Mental Health Composite Scale (MCS) score increased in the usual care group between randomization and 1 month, but decreased (worse health status) slightly at the 3‐month follow‐up. In the NPWT group, MCS score declined over time (*Table* 3).

**Table 3:** Short Form 12 Physical and Mental Health Composite Scale scores

	NPWT (*n* = 19)		Usual care (*n* = 21)		Total (*n* = 40)	
	*n*	Score*	*n*	Score*	*n*	Score*
Physical Composite Scale						
Baseline	16	28·9(10·6)	19	34·9(11·4)	35	32·1(11·3)
Month 1	14	29·8(10·1)	15	38·8(12·1)	29	34·5(11·9)
Month 3	14	30·4(11·9)	14	42·8(8·3)	28	36·6(11·9)
Mental Health Composite Scale						
Baseline	16	44·7(12·6)	19	42·2(11·2)	35	43·3(11·8)
Month 1	14	42·7(13·8)	15	48·6(11·7)	29	45·8(12·9)
Month 3	14	41·5(10·9)	14	47·1(12·1)	28	44·3(11·6)

* Values are mean(s.d.). NPWT, negative‐pressure wound therapy.

In both groups there was minimal change in BPI severity or interference, whereas VAS wound pain scores decreased over time. In the NPWT group, VAS pain at dressing changes peaked at 1 month, whereas in the usual care group it appeared to decrease over time (*Table* 4).

**Table 4:** Pain scores by randomized group and time point

	NPWT (*n* = 19)		Usual care (*n* = 21)		Total (*n* = 40)	
	*n*	Score*	*n*	Score*	*n*	Score*
BPI severity						
Baseline	16	4·4(2·8)	20	2·7(2·8)	36	3·5(2·9)
Week 2	15	3·7(2·8)	17	2·5(2·5)	32	3·0(2·7)
Month 1	14	3·5(3·4)	15	1·9(2·6)	29	2·7(3·1)
Month 3	14	4·3(2·9)	13	2·1(2·6)	27	3·2(2·9)
BPI interference						
Baseline	15	5·0(3·5)	16	2·8(3·6)	31	3·8(3·6)
Week 2	15	4·5(3·5)	17	3·3(3·3)	32	3·9(3·4)
Month 1	14	4·2(3·7)	15	2·3(2·8)	29	3·2(3·4)
Month 3	13	5·3(2·8)	15	2·7(3·4)	28	3·9(3·4)
VAS wound pain						
Baseline	15	51·2(32·1)	18	34·8(32·7)	33	42·2(33·0)
Week 2	13	32·4(33·4)	17	26·5(29·3)	30	29·1(30·7)
Month 1	13	27·1(31·1)	14	20·7(26·7)	27	23·8(28·5)
Month 3	12	30·8(27·2)	15	13·5(23·1)	27	21·1(26·0)
VAS pain at dressing changes†						
Week 2	13	22·3(25·5)	15	22·6(24·5)	28	22·5(24·5)
Month 1	13	26·5(32·8)	14	16·4(24·2)	27	21·2(28·5)
Month 3	11	18·1(20·2)	15	12·5(27·0)	26	14·8(24·1)

* Values are mean(s.d.).

† Not measured at baseline. NPWT, negative‐pressure wound therapy; BPI, Brief Pain Inventory; VAS, visual analogue scale.

#### *Clinical and adverse events*

A total of 1135 dressing changes were completed during the study (NPWT, 518; usual care, 617). The mean(s.d.) number of dressing changes for NPWT participants was 27·26(18·91), and that for usual care participants was 29·38(14·38).

Wound infection was observed, through clinical review against a predefined features list, in 17 participants (NPWT, 6; usual care, 11). In total, 13 participants had a wound infection reported at baseline; in nine the infection resolved and did not reoccur (NPWT, 3; usual care, 6), and in four the infection resolved but later reoccurred (NPWT, 2; usual care, 2). Four further participants developed an infection at some point during their postrandomization follow‐up (NPWT, 1; usual care, 3).

Duration of hospital stay data were collected using participant‐reported questionnaires. Eight participants in the NPWT group reported spending nights in hospital during the trial (median 29 (range 2–60) nights). Only one participant in the usual care group reported spending nights in hospital (10 nights).

Five serious adverse events were reported, two in the NPWT group from one participant, and three in the usual care group from two participants. All events were related to hospitalization (uncontrolled diabetes, 2; infected wound, 1; fall, 1; amputation due to osteomyelitis, 1), and only one (usual care group; infected foot wound) was reported as related to the reference wound. No events were deemed to be definitely related to treatment received, but three of the five events (amputation due to osteomyelitis, infected wound and one instance of uncontrolled diabetes) were possibly or probably related, all of which were expected. There were no unexpected serious adverse events.

Twenty‐eight non‐serious adverse events were reported. The majority (25) were related to the reference wound, and most (19) were reported as definitely related to treatment (strong evidence that the event was caused by NPWT device or usual care dressings). The most common event type was ‘other’ (12 events), which included events such as skin irritation (3), dressing falling off (3) and pump failure (8).

#### *Resource use and cost‐effectiveness analysis*

Participants in the NPWT group reported more hospital care and general practitioner (GP) visits, but fewer nurse visits (at surgery or home) in relation to the wound than those in the usual care group; this was reflected in the associated wound‐related costs of each group (*Table* 5). The mean(s.d.) total cost for the NPWT intervention was €13 096·20(10 137·48) compared with €1591·15(2492·28) for a patient under usual care (expressed in euros using a 2015 price level of £1 sterling = €1·38)^8^. A large proportion of the costs for patients in the NPWT group were attributable to hospital care: mean(s.d.) €10 639·80(10 428·66) *versus* €931·50(2517·12) for usual care. Conversely, primary and community care costs were lower for the NPWT group; for costs related to GP and nurse visits, NPWT cost a mean of €291·18 (95 per cent c.i. −611·34 to 27·60) less per patient in comparison with usual care.

**Table 5:** Total mean costs per patient based on all available patients up to 3‐month follow‐up

	NPWT		Usual care		Incremental cost† (€)
	*n*	Total cost (€)*	*n*	Total cost (€)*	
Combined resource use		13 096·20(10 137·48)		1591·15(2492·28)	11 505·06 (4569·36, 18 440·88)
Hospital outpatient clinic					
Doctor	15	497·86(590·07)	13	140·11(212·33)	357·75 (2·25, 713·24)
Nurse	15	651·99(1012·78)	13	398·72(984·86)	253·27 (−525·63, 1032·18)
Day‐case hospital visit	12	331·56(882·90)	14	213·14(797·50)	118·42 (−561·76, 798·59)
Nights as hospital inpatient	14	8672·98(9836·87)	11	450·54(1494·28)	8222·44 (2003·95, 14 440·93)
GP visit					
At GP practice	14	82·40(164·32)	13	42·03(79·87)	40·37 (−63·37, 144·11)
At home	14	35·05(75·00)	13	0	35·05 (−7·84, 77·96)
Nurse visit					
At GP practice	14	17·51(84·46)	12	122·65(228·33)	−105·14 (−232·32, 22·05)
At home	14	218·47(316·24)	12	399·76(484·28)	−181·29 (−507·74, 145·16)

* Mean(s.d.) costs converted from GBP to euros using the average yearly price level for 2015 (£1 sterling = €1·38)^8^.

† Cost for negative‐pressure wound therapy (NPWT) minus cost for usual care; values in parentheses are 95 confidence intervals. GP, general practitioner.

The baseline EQ‐5D‐3L™ score was lower (worse HRQoL) for participants receiving NPWT (mean(s.d.) 0·34(0·10) *versus* 0·54(0·08) in the usual care group). At 3 months, after adjustment for baseline utility, scores had improved: scores were 0·49(0·35) and 0·77(0·23) respectively. EQ‐5D‐3L™ data were missing for three participants in the NPWT group, so could not be included in the analyses. This, in combination with the small sample size, accounts for differences in the mean scores between the groups at baseline.

Participants who received NPWT had higher total NHS and healthcare costs, and also achieved lower QALY gains than those receiving usual care (complete case analysis: difference −0·007, 95 per cent c.i. −0·04 to 0·02). To account for the levels of missing data observed for EQ‐5D‐3L™, multiple imputation analysis was conducted; results were similar to those for the complete case analysis (difference −0·004, −0·03 to −0·02).

## Discussion

This pilot study has provided invaluable information on the feasibility of conducting a large‐scale trial of NPWT for the treatment of SWHSI.

After the opening of all sites to recruitment, the consistent rate of recruitment observed indicates that it is possible to recruit participants to a trial of NPWT *versus* usual care in this patient population. A full trial would, however, need to identify a sufficient number of recruiting sites. For example, based on the recruitment rate observed (2 patients per site per month), a minimum of 11 study sites would need to be opened to recruit 400 participants to a similar trial over an 18‐month period. Sample size calculations would also need to consider the impact of (and adjust accordingly for) the rates of participants allocated to but not subsequently receiving NPWT (2 of 19 in the present study) and usual care crossover rates (5 of 21 in this study).

The majority of the 40 participants had a wound located on the foot (24 patients, 60 per cent), probably because this patient group was more readily able to be recruited to this pilot feasibility study. Existing enthusiasm for NPWT may have produced a lack of equipoise at the study sites involved, with some health professionals being unwilling to include their patients in the study. Thus, some patient subgroups could not be recruited and are under‐represented in this sample. Careful engagement of surgeons in advance of a full trial would be required to ensure equipoise, and thereby promote recruitment. The nature of engagement would depend on whether a homogeneous or heterogeneous study population was proposed for inclusion in the trial.

The choice of study population for inclusion in a larger trial would need to be considered carefully, given the heterogeneity of the population of patients with SWHSI. Even within subpopulations, for example those with foot SWHSI, their heterogeneous nature must be considered. Future trials should consider how best to balance ease of recruitment, by including a heterogeneous population, and comparability of data, by focusing on a smaller subpopulation.

For this pilot feasibility study, the majority of participants were recruited in acute care settings, as this is where patients with complex wounds are routinely cared for initially. A future RCT may therefore benefit from targeting recruitment in acute care settings initially. The distribution of participants in acute and community settings may differ between individual localities, so before recruitment it would be necessary to ensure that appropriate recruitment strategies and pathways were identified and implemented at individual sites.

The most frequent reason for study ineligibility was patients previously or currently receiving NPWT on their SWHSI. A future trial should aim to recruit study sites where there is sufficient equipoise in treatment of patients with SWHSI; thus site evaluation before site inclusion would be essential. Additionally, before undertaking a full RCT, promotional activities with clinicians and research groups to engage surgeons and nurses within the study site would be valuable to ensure consistent and continued referrals into the study. Such work may also help to allay resistance to trial recruitment from certain clinical groups. Anecdotal evidence from the sites in this pilot study suggested some resistance to recruitment of patients by some surgeons, either because NPWT was used primarily for wound management, as opposed to healing, or due to surgeons' prior experience of NPWT, leading them to believe this treatment was inappropriate for use in a specific patient group. Given the small number of sites included in this pilot study, it is impossible to draw firm conclusions on whether resistance amongst specific clinical groups would be present in a larger RCT.

A participation rate of 65 per cent, participant‐reported questionnaire response rates in excess of 75 per cent, and a limited number of withdrawals demonstrate that eligible patients are willing to engage and commit to a RCT of NPWT *versus* usual care.

During the study, only two of the 40 participants were withdrawn from the trial, both due to amputation of the reference wound. Data collection did not continue for these participants, and the trial protocol was subsequently amended to reflect that clinical data would cease at the point of amputation. Participant‐reported and resource use data could continue to be collected, subject to continued consent, to capture the impact of limb amputation on patient HRQoL and costs incurred. A future trial should ensure that the process for continued data collection following amputation is clearly defined before commencing study recruitment.

The movement of patients between acute and community settings did not affect follow‐up completion, with follow‐up continuing as planned in the majority of cases. The research nurses did, however, note that on occasion it was impossible to assess the wound at follow‐up visits, primarily as a result either of community healthcare professionals (such as district nurses) having already attended and redressed the wound or of community healthcare professionals not attending at the agreed time for the visit, so that research data could not be collected in full.

In general, participants received NPWT delivery within 48 h of randomization; however, three participants did not receive NPWT within this time. Two participants did not receive NPWT at all, due to lack of intervention availability and potential for delays to discharge. The availability of NPWT devices should be established at sites from the outset of a future RCT to ensure timely delivery of the intervention.

As evidence for the effectiveness of specific wound dressings is limited, and often of low quality, the choice of dressings for use as a comparator within a trial setting requires consideration. Given the number of changes made to the types of dressing used for individual patients in routine care, and differences in guidelines and dressing availability in individual NHS trust settings, it is likely to be difficult to standardize to a single dressing. It may therefore be more pragmatic to consider permitting the use of any dressing, or a specific range of dressing types, in a future trial.

Treatment crossover was recorded for five of the 21 participants in the usual care group. In view of the impact that crossover may have in relation to estimates of effect, future trials should consider whether use of NPWT can be minimized or prevented in the usual care arm. If it cannot be prevented, appropriate statistical analysis should be used to account for this crossover.

Nurses involved in the study reported that they had received sufficient training within their individual institutions to apply NPWT confidently. They noted, however, that clinical colleagues were not always confident in using NPWT, which corresponds to findings from a recent qualitative study^16^. A future trial would therefore need to ensure that NPWT training was provided to all staff who may administer trial treatment as part of the study.

This study has demonstrated that collection of data regarding future potential primary and secondary clinical outcome measures is feasible; no major obstacles were encountered. Given the limited number of participants involved in this study, there was insufficient power to enable any true treatment effect of NPWT to be assessed, and, indeed, this was not the purpose of the study. The associations between co‐variables and wound healing obtained using Cox proportional hazards regression should be interpreted with caution. A full RCT is required to assess fully the clinical and cost‐effectiveness of NPWT as a treatment for patients with SWHSI.

Observational work (I. Chetter *et al*., unpublished data, 2018) preceding this pilot RCT identified a median time to healing for all patients of 86 days. As a result it was deemed that follow‐up of 90 days might be sufficient to observe wound healing. As only ten (25 per cent) of the 40 wounds healed during the course of this trial, a 3‐month follow‐up is probably inadequate for reliable investigation and comparison of healing rates. Follow‐up for a minimum duration of 6 months would therefore need to be considered for a larger trial.

The difference in time to healing found in this pilot feasibility trial and a previous cohort study (I. Chetter *et al*., unpublished data, 2018) may be explained by the greater proportion of participants with foot and leg wounds recruited to the trial (usually secondary to diabetes). The differences in healing trajectory for specific wound types and the complexities associated with wound healing in people with diabetes^17^ may explain these lower healing rates.

The number of nights spent as an inpatient differed between trial arms, and may be explained by regional arrangements for the supply of NPWT to NHS patients following discharge from the acute setting. These arrangements may be specific to the participating sites involved in the study, and not necessarily indicative of NHS‐wide processes. To mitigate the impact of regional arrangements on duration of inpatient stay, local discharge arrangements in relation to patients using NPWT and availability of NPWT within community settings should be discussed as part of preliminary engagement with potential recruitment sites. This will ensure that sufficient provisions are in place at all recruiting sites and facilitate homogenization of NPWT delivery across study sites. This is critical to ensure the smooth delivery of a larger RCT and to prevent significant differences in treatment delivery and cost between study centres.

Assessment of wound measurements indicated that ruler measurements, both with and without adjustment, overestimate wound size compared with wound tracings. Wound tracings are therefore recommended as the primary source for wound measurement in a larger RCT.

This pilot trial has demonstrated the feasibility of blinded outcome assessment of a single photograph in the context of NPWT for SWHSI; the logistics of reviewing the full photograph set for each patient were too complex. As a result, it was not possible to derive time to healing through the blinded outcome assessment.

Blinded and unblinded investigator assessment in relation to wound healing agreed in seven of eight cases, although blinded assessment of healing was often hampered by the quality of the wound photograph. Assessors correctly identified NPWT allocation more frequently than usual care; however, as overall identification of the correct allocation was observed in only about one‐third of cases (15 of 40), blinded assessment can be deemed to be feasible and appropriate within a future RCT. Previous venous leg ulcer studies^18^ ^19^ have used blinded outcome assessment to assess wound‐related outcomes (time to wound healing, debridement status). These studies demonstrated that remote blinded outcome assessment can be completed successfully in wound‐related research assessing healing outcomes. A robust photography procedure should be implemented to ensure consistency of imaging completion and return throughout the study, and reliability of time‐to‐healing as an outcome measure.

This pilot study has identified key elements and recommendations for recruitment, data collection, outcome measurement, trial design and safety reporting that should be considered for a larger RCT to investigate the clinical and cost‐effectiveness of NPWT *versus* usual care in patients with SWHSI. A full RCT will aim to obtain more definitive evidence and so reduce the current uncertainty surrounding NPWT efficacy and cost‐effectiveness.

## Supporting information


**Appendix S1.** Clinical assessment of wound infection
**Table S1.** Wound pain collected using weekly text messages by randomized group and time point
**Table S2.** Coefficients from the Cox proportional hazards regression models for time to healing
**Table S3.** Wound area from tracings by randomized group over time

## References

[1] NHS Confederation . *Key Statistics on the NHS*; 2016 http://www.nhsconfed.org/resources/key-statistics-on-the-nhs [accessed 11 June 2016].

[2] Mees J , Mardin WA , Senninger N , Bruewer M , Palmes D , Mees ST . Treatment options for postoperatively infected abdominal wall wounds healing by secondary intention. Langenbecks Arch Surg 2012; 397: 1359–1366. 22875224 10.1007/s00423-012-0988-7

[3] Vermeulen H , Ubbink DT , Goossens A , de Vos R , Legemate DA . Systematic review of dressings and topical agents for surgical wounds healing by secondary intention. Br J Surg 2005; 92: 665–672. 15912490 10.1002/bjs.5055

[4] Norman G , Dumville JC , Mohapatra DP , Owens GL , Crosbie EJ . Antibiotics and antiseptics for surgical wounds healing by secondary intention. Cochrane Database Syst Rev 2016; (3)CD011712. 10.1002/14651858.CD011712.pub2 PMC6599835 27021482

[5] National Institute for Health and Care Excellence (NICE) . Surgical Site Infections: Prevention and Treatment. *Clinical Guideline CG74*. NICE: London, 2008. 31211539

[6] Banwell PE , Musgrave M . Topical negative pressure therapy: mechanisms and indications. Int Wound J 2004; 1: 95–106. 16722882 10.1111/j.1742-4801.2004.00031.x PMC7951556

[7] Dumville JC , Owens GL , Crosbie EJ , Peinemann F , Lui Z . Negative pressure wound therapy for treating surgical wounds healing by secondary intention. Cochrane Database Syst Rev 2015; (6)CD011278. 10.1002/14651858.CD011278.pub2 PMC10960640 26042534

[8] UKForex Limited. *Yearly Average Rates* http://www.ofx.com/en-gb/forex-news/historical-exchange-rates/yearly-average-rates/ [accessed 7 December 2016].

[9] Ashby RL , Dumville JC , Soares MO , McGinnis E , Stubbs N , Torgerson DJ *et al* A pilot randomised controlled trial of negative pressure wound therapy to treat grade III/IV pressure ulcers [ISRCTN69032034]. Trials 2012; 13: 119. 22839453 10.1186/1745-6215-13-119 PMC3533804

[10] http://clinicaltrials.gov. *Negative Pressure Wound Therapy for the Treatment of Chronic Pressure Wounds (NPWT)*; 2016 http://clinicaltrials.gov/ct2/show/NCT00691821 [accessed 11 June 2016].

[11] Arundel C , Buckley H , Clarke E , Cullum N , Dixon S , Dumville J *et al* Negative pressure wound therapy *versus* usual care for Surgical Wounds Healing by Secondary Intention (SWHSI trial): study protocol for a randomised controlled pilot trial. Trials 2016; 17: 535. 27821142 10.1186/s13063-016-1661-1 PMC5100221

[12] Kundin JI . A new way to size up wounds. Am J Nurs 1989; 89: 206–207. 2916597

[13] Taylor RJ . ‘Mouseyes’: an aid to wound measurement using a computer. J Wound Care 1997; 6: 123–126. 9256708 10.12968/jowc.1997.6.3.123

[14] Cleeland CS , Ryan KM . Pain assessment: global use of the Brief Pain Inventory. Ann Acad Med Singapore 1994; 23: 129–138. 8080219

[15] Bland MJ , Altman D . Statistical methods for assessing agreement between two methods of clinical measurement. Lancet 1986; 1: 307–310. 2868172

[16] McCaughan D , Sheard L , Cullum N , Dumville J , Chetter I . Patients' perceptions and experiences of living with a surgical wound healing by secondary intention: a qualitative study. Int J Nurs Stud 2018; 77: 29–38. 29031127 10.1016/j.ijnurstu.2017.09.015 PMC5744862

[17] Armstrong DG , Lavery LA ; Diabetic Foot Study Consortium . Negative pressure wound therapy after partial diabetic foot amputation: a multicentre, randomised controlled trial. Lancet 2005; 366: 1704–1710. 16291063 10.1016/S0140-6736(05)67695-7

[18] Dumville JC , Worthy G , Bland JM , Cullum N , Dowson C , Iglesias C *et al*; VenUS II team . Larval therapy for leg ulcers (VenUS II): randomised controlled trial. BMJ 2009; 338: b773. 19304577 10.1136/bmj.b773 PMC2659858

[19] Ashby RL , Gabe R , Ali S , Saramago P , Chuang LH , Adderley U *et al* VenUS IV (Venous leg Ulcer Study IV) – compression hosiery compared with compression bandaging in the treatment of venous leg ulcers: a randomised controlled trial, mixed‐treatment comparison and decision‐analytic model. Health Technol Assess 2014; 18: 1–293. 10.3310/hta18570 PMC4781202 25242076

