# Supplementary material for: Pilot feasibility randomized clinical trial of negative‐pressure wound therapy versus usual care in patients with surgical wounds healing by secondary intention
Source: BJS Open. 2018 Apr 23;2(3):99–111. doi: 10.1002/bjs5.49 (PMC5989956; doi:10.1002/bjs5.49)
Supplement: Supplementary file 1 — Appendix S1. Clinical assessment of wound infection Table S1. Wound pain collected using weekly text messages by randomized group and time point Table S2. Coefficients from the Cox proportional hazards regression models for time to healing Table S3. Wound area from tracings by randomized group over time [file BJS5-2-99-s001.docx]

**BJS5_49**

**Pilot feasibility randomized clinical trial of negative-pressure wound therapy *versus* usual care in patients with surgical wounds healing by secondary intention**

**C. Arundel, C. Fairhurst, B. Corbacho-Martin, H. Buckley, E. Clarke, N. Cullum, S. Dixon, J. Dumville, A. Firth, E. Henderson, K. Lamb, E. McGinnis, A. Oswald, P. Saramago Goncalves, M. O. Soares, N. Stubbs and I. Chetter**

**Appendix S1** Clinical assessment of wound infection

**In your clinical opinion is this wound currently infected?**

Yes No Don’t know

**If ‘Yes’, please indicate which of the following features are present:**

|  | Abscess or other evidence of infection found during re-operation, by radiology or histopath examination |  | Antibiotics prescribed for surgical site infection |
| --- | --- | --- | --- |
|  | Aspirated fluid/swab of surgical site yields organisms and pus cells are present |  | Clinicians diagnosis |
|  | Fever (temperature 38°C or more) |  | Heat |
|  | Incision spontaneously dehisces or opened by surgeon |  | Localised pain and tenderness |
|  | Localised swelling |  | Purulent drainage |
|  | Redness |  |  |

**Table S1** Wound pain collected using weekly text messages by randomized group and time point

| **Weekly text message pain scores**  N received/N sent (%)  Mean (SD) | **NPWT**  **(n=10)** | **Usual Care**  **(n=10)** | **Total**  **(n=20)** |
| --- | --- | --- | --- |
| **Week 1** | 6/13 (46.2)  5.0 (1.9) | 9/13 (69.2)  3.3 (3.4) | 15/26 (57.7)  4.0 (3.0) |
| **Week 2** | 10/13 (76.9)  4.9 (3.2) | 9/12 (75.0)  3.0 (3.0) | 19/25 (76.0)  4.0 (3.2) |
| **Week 3** | 9/12 (75.0)  3.4 (2.8) | 8/10 (80.0)  2.6 (3.0) | 17/22 (77.3)  3.1 (2.8) |
| **Week 4** | 9/11 (81.8)  3.9 (3.0) | 9/9 (100.0)  2.2 (2.4) | 18/20 (90.0)  3.1 (2.8) |
| **Week 5** | 10/11 (90.9)  3.6 (3.2) | 6/8 (75.0)  2.2 (2.5) | 16/19 (84.2)  3.1 (3.0) |
| **Week 6** | 9/11 (81.8)  2.6 (3.5) | 7/8 (87.5)  2.7 (2.9) | 16/19 (84.2)  2.6 (3.1) |
| **Week 7** | 7/8 (87.5)  3.6 (3.9) | 7/7 (100.0)  2.1 (2.7) | 14/15 (93.3)  2.9 (3.3) |
| **Week 8** | 6/7 (85.7)  3.7 (3.5) | 5/7 (71.4)  1.4 (2.1) | 11/14 (78.6)  2.6 (3.0) |
| **Week 9** | 5/7 (71.4)  2.8 (2.6) | 7/7 (100.0)  1.4 (1.9) | 12/14 (85.7)  2.0 (2.2) |
| **Week 10** | 5/7 (71.4)  3.0 (2.1) | 6/6 (100.0)  1.2 (1.6) | 11/13 (84.6)  2.0 (0.2) |
| **Week 11** | 5/5 (100.0)  3.2 (2.4) | 7/7 (100.0)  0.9 (1.2) | 12/12 (100.0)  1.8 (2.1) |
| **Week 12** | 5/5 (100.0)  2.4 (2.4) | 6/6 (100.0)  1.0 (1.5) | 11/11 (100.0)  1.6 (2.0) |

**Table S2** Coefficients from the Cox proportional hazards regression models for time to healing

| **Model 1: Predictor** | **HR (95% CI)** | **p-value** |
| --- | --- | --- |
| **Allocation^a^:** NPWT | 9.10 (1.30, 63.62) | 0.03 |
| **Wound size, cm^2^ (continuous)** | 0.96 (0.92, 1.00) | 0.03 |
| **Duration of wound, days (continuous)** | 1.06 (1.03, 1.10) | 0.001 |
| **Contamination level of surgery^b^**  Clean/contaminated  Contaminated | 2.15 (0.40, 11.64)  0.11 (0.00, 2.25) | 0.37  0.15 |
| **Infection^c^:** Yes | 2.10 (0.33, 13.51) | 0.44 |
|  |  |  |
| **Model 2: Predictor** | **HR (95% CI)** | **p-value** |
| **Allocation^a^:** NPWT | 9.56 (1.30, 70.30) | 0.03 |
| **Wound size, cm^2^ (continuous)** | 0.95 (0.91, 1.00) | 0.03 |
| **Duration of wound, days (continuous)** | 1.06 (1.03, 1.10) | 0.001 |
| **Contamination level of surgery^b^**  Clean/contaminated  Contaminated | 2.18 (0.41, 11.64)  0.09 (0.00, 2.64) | 0.36  0.17 |
| **Infection^c^:** Yes | 2.13 (0.34, 13.34) | 0.42 |
| **History of SWHSI^d^:** Yes* | 1.30 (0.19, 8.78) | 0.27 |
|  |  |  |
| **Model 3: Predictor** | **HR (95% CI)** | **p-value** |
| **Allocation^a^:** NPWT | 5.19 (0.75, 36.05) | 0.10 |
| **Wound size, cm^2^ (continuous)** | 0.95 (0.91, 1.00) | 0.04 |
| **Duration of wound, days (continuous)** | 1.05 (1.01, 1.08) | 0.01 |
| **Contamination level of surgery^b^**  Clean/contaminated  Contaminated | 4.30 (0.60, 30.73)  0.29 (0.02, 4.70) | 0.15  0.38 |
| **Infection^c^:** Yes | 1.52 (0.21, 10.78) | 0.68 |
| **Location of SWHSI^e^:** Foot | 0.17 (0.02, 1.31) | 0.09 |
|  |  |  |
| **Model 4: Predictor** | **HR (95% CI)** | **p-value** |
| **Allocation^a^:** NPWT | 9.31 (1.22, 70.94) | 0.03 |
| **Wound size, cm^2^ (continuous)** | 0.96 (0.93, 1.00) | 0.05 |
| **Duration of wound, days (continuous)** | 1.06 (1.02, 1.10) | 0.001 |
| **Contamination level of surgery^b^**  Clean/contaminated  Contaminated | 1.94 (0.39, 9.71)  0.11 (0.00, 2.43) | 0.42  0.16 |
| **Infection at any point (time-varying)**  Yes | 0.99 (0.11, 8.94) | 1.00 |
| Reference category: ^a^ Usual Care; ^b^ contaminated; ^c^ no infection at baseline; ^d^ no history of SWHSI prior to reference wound (assumes history unknown is no history); ^e^ area of the body other than the foot; *assuming Don’t know = No | | |

**Table S3** Wound area from tracings by randomized group over time

| **Wound area, cm^2^**  N, Median (min, max) | **NPWT**  **(n=19)** | **Usual Care**  **(n=21)** | **Total**  **(n=40)** |
| --- | --- | --- | --- |
| **Baseline** | 17, 13.5 (1.5, 67.1) | 21, 15.2 (1.4, 59.5) | 38, 14.4 (1.4, 67.1) |
| **Week 1** | 10, 10.6 (2.3, 51.5) | 6, 13.5 (0.2, 37.1) | 16, 11.2 (0.2, 51.5) |
| **Week 2** | 10, 7.9 (0.1, 59.9) | 11, 6.55 (0.0, 52.7) | 21, 6.55 (0.0, 59.9) |
| **Week 3** | 12, 10.3 (0.5, 44.1) | 11, 9.89 (0.0, 29.9) | 23, 13.93 (13.27) 9.89 (0.0, 44.1) |
| **Week 4** | 11, 6.8 (0.3, 67.1) | 10, 5.6 (0.0, 54.8) | 21, 6.2 (0.0, 67.1) |
| **Week 5** | 9, 2.6 (0.1, 46.1) | 14, 5.4 (0.0, 33.3) | 23, 4.5 (0.0, 46.1) |
| **Week 6** | 10, 6.2 (0.0, 31.9) | 13, 5.9 (0.0, 53.2) | 23, 5.9 (0.0, 53.2) |
| **Week 7** | 10, 1.1 (0.0, 20.8) | 9, 3.8 (0.4, 27.6) | 19, 2.0 (0.0, 27.6) |
| **Week 8** | 16, 4.3 (0.0, 58.7) | 9, 1.2 (0.3, 22.2) | 25, 1.5 (0.0, 58.7) |
| **Week 9** | 7, 0.9 (0.0, 27.6) | 9, 2.3 (0.5, 10.3) | 16, 2.1 (0.0, 27.6) |
| **Week 10** | 10, 7.6 (0.0, 36.9) | 9, 3.9 (0.3, 15.8) | 19, 6.1 (0.0, 36.9) |
| **Week 11** | 7, 1.1 (0.0, 22.8) | 9, 5.0 (6.2)  2.7 (0.1, 17.7) | 16, 5.9 (7.6)  1.9 (0.0, 22.8) |
| **Week 12** | 6, 12.3 (0.6, 26.6) | 13, 2.8 (0.0, 41.9) | 19, 3.8 (0.0, 41.9) |
